# Commentary: *SPTBN5*, encoding the βV-spectrin protein, leads to a syndrome of intellectual disability, developmental delay, and seizures

**DOI:** 10.3389/fnmol.2022.1017684

**Published:** 2022-09-27

**Authors:** Liedewei Van De Vondel, Jonathan De Winter, Jonathan Baets

**Affiliations:** ^1^Department of Translational Neurosciences, Faculty of Medicine and Health Sciences, University of Antwerp, Edegem, Belgium; ^2^Laboratory of Neuromuscular Pathology, Institute Born-Bunge, University of Antwerp, Antwerp, Belgium; ^3^Department of Neurology, NeuroMuscular Reference Center, Antwerp University Hospital, Edegem, Belgium

**Keywords:** *SPTBN5*, whole exome sequencing, spectrin, intellectual disability (ID), rare diseases (RD)

## Introduction

The spectrin complex is a major component of the cytoskeleton in all cells. *SPTA1* and *SPTB* are expressed in erythrocytes, while *SPTAN1, SPTBN1, SPTBN2, SPTBN4* and *SPTBN5* show a more ubiquitous expression pattern. Mendelian disease-gene links are increasingly being recognized for the spectrin genes, with *SPTAN1, SPTBN1, SPTBN2* and *SPTBN4* linked to neurological diseases such as developmental and epileptic encephalopathy (DEE), ataxia, hereditary spastic paraplegia, and hereditary motor neuropathy. This wide range of associated phenotypes speaks to the critical nature of the spectrin complex in the nervous system.

Khan et al. (2022) correctly addressed the high likelihood that variants in *SPTBN5* would also result in neurological phenotypes. They present four variants, p.His89Pro, p.Tyr311Ter, p.Asn2937Tyr and p.Glu3262Lys respectively, as the cause of syndromic intellectual disability, developmental delay and seizures in four families. They report on the predicted molecular effects of the respective variants through *in silico* protein modeling.

## Subsections relevant for the subject

Despite the importance of reporting novel disease-gene links to improve genetic diagnostic rate in patients, the study by Khan et al. raises important concerns.

Some confusion arose with the variant state described as homozygous in the introduction, while the results section mentions heterozygous *de novo* mutations. The Sanger Sequencing traces in Figure 1B indeed shows heterozygous variants.

More crucially however, several concerns can be raised related to the level of genetic evidence presented for each *SPTBN5* variant. Firstly, the variant filtering strategy used with a Minor Allele Frequency (MAF) cut-off of 1% in the healthy population is not in accordance with the severity and age-of-onset of the described phenotypes (Gudmundsson et al., 2022). In family B the p.Glu3262Lys variant is identified with a GnomAD frequency of 0.0001, corresponding to an allele count of 24. In family D the p.Asn2937Tyr variant corresponds to an allele count of 1,042 and even 4 homozygotes in the healthy population – even when only evaluating the cohort excluded from having a neurological condition in GnomAD v2.1.1 (Karczewski et al., 2020). It is hard to reconcile this markedly high prevalence in the healthy population with a definite Mendelian disease-gene link for these specific variants in such a rare disorder, even taking into account the *de novo* nature of the variants.

The authors propose a haploinsufficiency mechanism leading to pathogenicity for the p.Tyr311Ter mutation in family C. Although this specific mutation only has a GnomAD allele count of 2, loss-of-function (LoF) variants in *SPTBN5* overall are highly frequent in the population, with as many as 190 homozygous LoF variants and more than 9000 heterozygous LoF variants. This is corroborated by the pLi score of 0, supporting the notion that *SPTBN5* is a gene that is highly tolerant to LoF (Karczewski et al., 2020).

In addition, in the clinical reports of the four families several crucial elements are missing, and other elements are confusing, possibly due to factual errors. While Figure 1 shows family B with two affected brothers, Table 1 and the results section describe a 15-year-old female in that family with no report of an affected sibling. The same inconsistency can be found for family C for which Figure 1 shows a female index patient and Table 1 and the results section mentions a 14-year-old male.

Based on literature the authors correctly state that disorders of the neuronal spectrin family share common features, most importantly neurodevelopmental disorders including epilepsy. Nevertheless, in their discussion they systematically fail to include *SPTAN1*, encoding α-II-spectrin, although this gene is highly associated with DEE (Marco Hernández et al., 2021). Furthermore, the description of the epilepsy phenotype is vague (“low rates of seizures compared to *SPTBN1* and *SPTBN4*”) and does not really allow to translate the findings toward clinical practice.

Finally, in the discussion the authors only briefly mention cerebral MR imaging in their cohort and do not mention presence (or absence) of corpus callosum atrophy, other morphological changes or any other (acquired) cerebral pathologies that could be pertinent to the description of these cases. These should be addressed given the relevance in patients with suspected DEE.

## Discussion

To conclude, the evidence to support the pathogenicity of the p.Tyr311Ter, p.Asn2937Tyr and p.Glu3262Lys variants in families B, C and D is not sufficient. With a GnomAD allele count of 1, the p.His89Pro variant in family A might still be the cause of the described phenotype; however confirmation of this finding in other patients and families is necessary. Overall, a loss-of-function mechanism is very unlikely for *SPTBN5* given the conservancy levels and common occurrence of LoF mutations in the healthy population. A dominant (or recessive) gain-of-function mechanism for *SPTBN5* is still possible however and certainly deserves further study.

## Author contributions

LV and JD: conceptualization and writing–original draft preparation. JB: critical revision of the manuscript. All authors have read and agreed to the published version of the manuscript.

## Funding

JB is supported by a Senior Clinical Researcher mandate of the Research Fund - Flanders (FWO) under grant agreement No1805021N. LV is supported by a predoctoral fellowship of the FWO under grant agreement No11F0921N. JD is supported by the Goldwasser-Emsens fellowship.

## Conflict of interest

The authors declare that the research was conducted in the absence of any commercial or financial relationships that could be construed as a potential conflict of interest.

## Publisher's note

All claims expressed in this article are solely those of the authors and do not necessarily represent those of their affiliated organizations, or those of the publisher, the editors and the reviewers. Any product that may be evaluated in this article, or claim that may be made by its manufacturer, is not guaranteed or endorsed by the publisher.
